# Knockdown of MVK does not lead to changes in NALP3 expression or activation

**DOI:** 10.1186/s12950-015-0048-5

**Published:** 2015-01-31

**Authors:** Fulvio Celsi, Elisa Piscianz, Maurizio Romano, Sergio Crovella

**Affiliations:** Institute for Maternal and Child Health – IRCCS “Burlo Garofolo”, via dell’Istria 65/1, 34137 Trieste, Italy; Department of Life Sciences, University of Trieste, Via A. Valerio 28, 34127 Trieste, Italy

**Keywords:** Immunology, Inflammation, Autoimmunity, Mevalonate kinase deficiency

## Abstract

**Background:**

Mutations in the *Mevalonate Kinase* gene (*MVK*) are causes of a rare autoinflammatory disease: Mevalonate Kinase Deficiency and its more acute manifestation, Mevalonic Aciduria. The latter is characterized, among other features, by neuroinflammation, developmental delay and ataxia, due to failed cerebellar development or neuronal death through chronic inflammation. Pathogenesis of neuroinflammation in Mevalonate Kinase Deficiency and Mevalonic Aciduria has not yet been completely clarified, however different research groups have been suggesting the inflammasome complex as the key factor in the disease development. A strategy to mimic this disease is blocking the mevalonate pathway, using HMG-CoA reductase inhibitors (Statins), while knock-out mice for Mevalonate Kinase are non-vital and their hemyzygous (i.e only one copy of gene preserved) littermate display almost no pathological features.

**Findings:**

We sought to generate a murine cellular model closely resembling the pathogenic conditions found in vivo, by direct silencing of Mevalonate Kinase gene. Knockdown of Mevalonate Kinase in a murine microglial cellular model (BV-2 cells) results in neither augmented NALP3 expression nor increase of apoptosis. On the contrary, statin treatment of BV-2 cells produces an increase both in Mevalonate Kinase and NALP3 expression.

**Conclusions:**

MKD deficiency could be due or affected by protein accumulation leading to NALP3 activation, opening novel questions about strategies to tackle this disease.

**Electronic supplementary material:**

The online version of this article (doi:10.1186/s12950-015-0048-5) contains supplementary material, which is available to authorized users.

## Introduction

Mevalonate Kinase is a key enzyme in the cholesterol biosynthetic pathway and is responsible for converting mevalonic acid to mevalonate-5-phosphate, an early intermediate in sterol and isoprenoid synthesis. Mutations in the *MVK* gene (12q24) encoding Mevalonate Kinase enzyme (MVK) cause Mevalonate Kinase Deficiency (MKD, OMIM #260920) and its more severe form, Mevalonic Aciduria (MA, OMIM #610377). The latter is characterized by recurrent attacks of fever, developmental delay, ataxia, dysmorphic features, failure to thrive, cataracts, and retinal dystrophy. The prognosis is poor: few patients survive until adolescence and more than 50% of them succumb to an inflammatory crisis during the first years of life [1]. One of the characteristics of this disease is cerebellar atrophy, due to neuronal apoptosis [2].

Although the genetic defects are well known and characterized, the pathogenic mechanism remains elusive, and treatment of the disease remains mainly supportive, with poor efficacy. For an evidence-based therapy, it is crucial to determine the exact pathogenic mechanisms occurring in the disease. Animals or cell-based models are thus necessary; in the past a mouse model of MVK deficiency was generated [3] however it was unable to correctly represent the pathology. Then a cellular model have been developed, where the mevalonate pathway is pharmacologically targeted using HMG-CoA blockers (statins), mimicking some of the inflammatory features observed in cells from patients [4]. Using this strategy, our group and other authors have found that, in different cell lines, NALP3 inflammasome activation is responsible for initiating inflammation [4-6]. This multi-molecular complex, constituted by NALP3, ASC and Caspase-1, cleaves pro-IL-1β to release its active form, starting inflammation [7]. Accordingly, NALP3 activation could represent a possible starting point for MA pathogenic progression. A number of issues need to be clarified, such as how the inflammasome pathway is incorrectly activated in cells presenting the pathological mutations. In the attempt to better mimic MA pathology we developed a novel murine cell model in which *MVK* expression is specifically down-regulated, using the silencing RNA (siRNA) technology. We then compared the results obtained by siRNA transfection with those achieved with Lovastatin and LPS treatment, in order to determine the better model suitable for mimicking the pathologic condition.

## Materials and methods

### BV-2 cell culture, transfection and treatment

During febrile attacks, MVK deficient patients experience an acute phase response showing increased plasma levels of IL-1β, IL-6 and TNF-α, and elevated urinary leukotriene E4 and neopterin excretion [8,9]. These observations have suggested that activation of macrophages might play a crucial pathogenic role, during as well as between attacks [10]. Evidences suggest that also the nervous system is involved in the most severe forms of MKD and that microglia might be activated consequently triggering neuroinflammation [11]. For these reasons, we used the murine microglial BV-2 cell line for the MVK RNAi experiments.

The microglial cell line BV-2 was maintained in RPMI medium (Euroclone) supplemented with 10%FBS, Pen/Strep. All experiments were conducted in passages between 2 and 8-10 to avoid effects due to cellular senescence. siRNA transfection was performed using Lipofectamine 2000 (LifeTechnologies) following manufacturer’s instructions. siRNA was obtained from Origene, (cat#SR413228), three different siRNA duplexes named 8a,8b,8c were used. Only 8b and 8c were able to produce significant reduction in MVK expression. Lovastatin was dissolved in 99% Ethanol at a concentration of 10mM and diluted to working concentration (50 μM) in cellular media. LPS (Sigma) from E. Coli 055:B5 has been dissolved in water in a stock concentration of 1 mg/ml and diluted to working concentration of 1 μg/ml. Lovastatin was applied for 24h and at the final 4h LPS was added in combined experiments Lova + LPS.

### Real-time PCR

Total cellular RNA was extracted using TRI-reagent (Sigma) following manufacturer’s instructions and retro-transcribed using High-Capacity cDNA Reverse transcription kit (Applied Biosystems). To quantify mRNA, Taq-man probes for MVK, NALP3 and β-Actin (as calibrator), were used (Applied Biosystems). Raw fluorescent data were converted to fold-increase using the software LinRegPCR [12] corrected for the analysis of hydrolysis probe [13], using the ΔΔCt method. In these calculations, β-Actin was used as internal standard and “scrambled” samples used as reference.

### Western blot

Cells were lysed in Lysis Buffer (LB: 300mM NaCl, 1%NP-40, 50mM Tris-HCl pH 7.4, 1mM EDTA) for 15 minutes in ice. Proteins were then quantified, using Bio-Rad Bradford protein assay (Bio-Rad), loaded onto a 4-20% gradient gel (Bio-rad) and electrophoretically separated. Subsequently proteins were transferred to nitrocellulose membrane (Bio-rad) incubated with NALP3 antibody (1:500, Abcam) and HSP90 antibody as a loading control (1:4000, Santa Cruz). Membranes were then incubated with secondary antibody HRP-conjugated and then revealed with Clarity ECL substrate (Bio-Rad) visualized with ChemiDoc™ MP imager (Bio-Rad). NALP3 protein expression was evaluated by densitometric analysis using ImageJ software (NIH). NALP3 mean optical density (O.D.) was then normalized to HSP90 average optical density and then the normalized O.D. from cells treated with scrambled siRNA (SCR) was set as 1 and other treatment related to SCR.

### Cell death analysis

The programmed cell death of BV-2 was evaluated by flow cytometry using Annexin V (A) and Propidium Iodide (PI) staining. Briefly, 48 hours after stimulation, BV-2 cells were harvested from the plates with a cell scraper and washed with PBS. After harvesting, BV-2 cells were stained for 15 minutes with FITC-conjugated A and PI (Annexin V-FITC Apoptosis Detection Kit, Immunostep) following the manufacturer’s indications.

Fluorescence was acquired with a CyAn ADP analyzer and Summit software (Beckman Coulter, Brea, CA), then analyzed with FlowJo software (version 7.6, Treestar Inc., Ashland, OR). Debris was excluded from the plot based on the scatter. Subsequently, apoptosis was characterized on the basis of the positivity to Annexin V and/or PI.

### Statistic

For real-time PCR analysis, experiments were performed in 5 replicates and analysis of each experiment was executed in triplicate wells. For Western Blot, experiments were made in 4 replicates. For Cell Death analysis, experiments were performed in 3 replicates. ANOVA followed by Bonferroni correction was used to determine significant changes between the samples, when p < 0.05. Statistical analysis was performed using software Prism™ (Graphpad).

## Results

In order to obtain an in vitro model of MVK deficiency, the effects of silencing Mevalonate Kinase expression with siRNA on the activation of the inflammasome pathway were detected.

**Figure 1:**
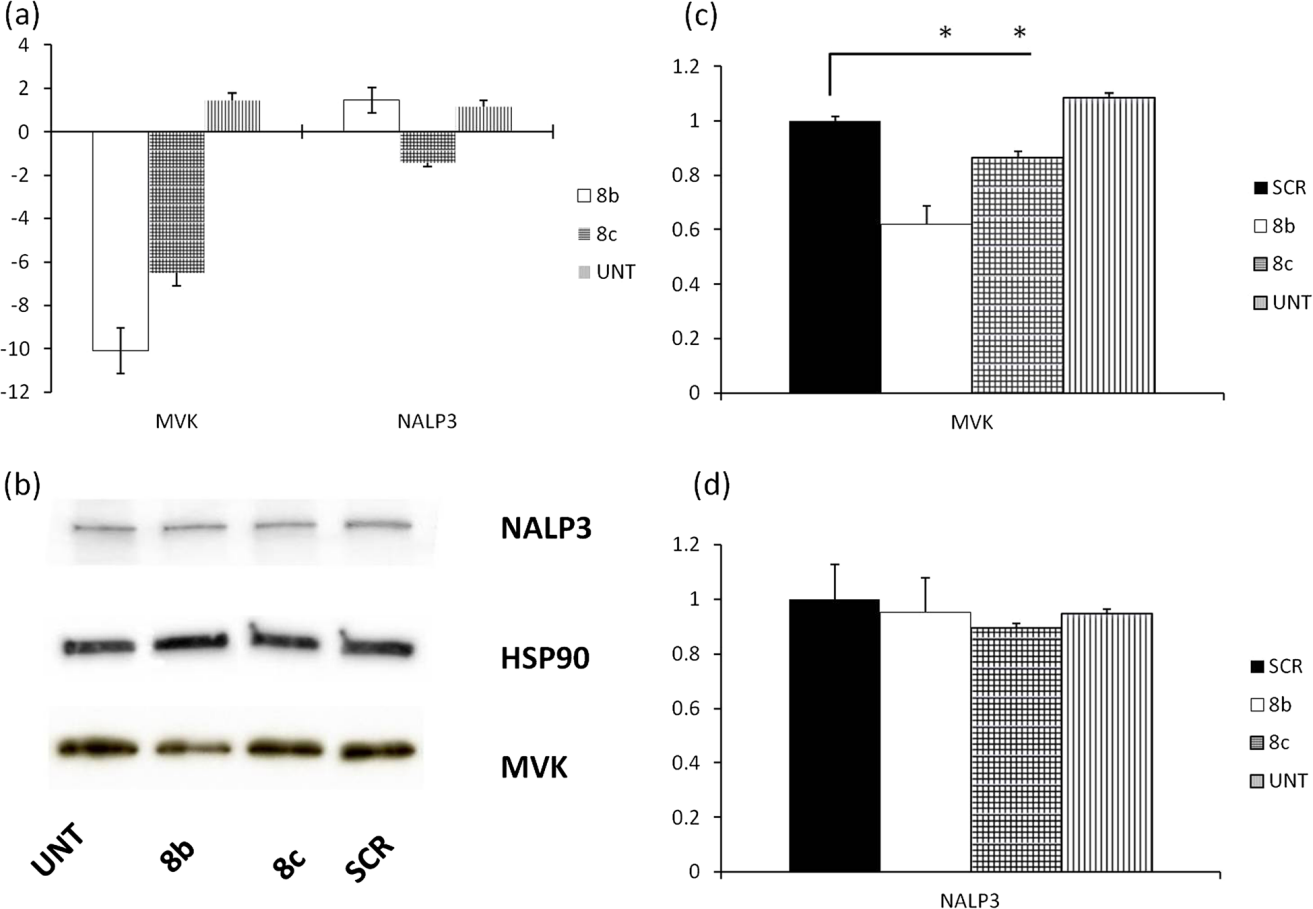
**Analysis of inflammasome components expression after siRNA treatment (a) ΔΔCt analysis of mRNA expression.** Left MVK expression, right NALP3 expression. mRNA expression in cells trasfected with “scrambled” sequences were considered as reference. * = p < 0.05 in comparison with UNT, i.e. untreated condition, cells subjected to transfection but without any siRNA **(b)** Representative western blot: UNT cells subjected to transfection but without any siRNA. **(c)** Optical density calculation of MVK protein levels. Scrambled (SCR) condition was set as 1. * = p < 0.05**(d)** Optical density calculation of NALP3 expression.

We transfected BV-2 cell line with two different *MVK* specific siRNA sequences (8b and 8c) and evaluated *MVK* mRNA and protein expression (Figure 1). After different time-course experiments (data not shown) we found that at 72h post-transfection MVK mRNA was reduced 10-fold in cells treated with 8b siRNA and 6-fold with 8c siRNA (Figure 1a, left). Correspondingly we found 40% reduction in MVK protein in 8b cells and 20% reduction in 8c cells (Figure 1b and c). In parallel, we evaluated the expression of NALP3, a key component of inflammasome pathway. Interestingly we found that neither mRNA levels nor protein levels were influenced by MVK silencing (Figure 1a, right, b and d). In addition we measured caspase-1 activity to confirm that inflammasome platform was not activated. We found that siRNA treatment did not significantly increase caspase-1 activity 72h after transfection compared to untreated cells (Additional file 1: Figure S1a). Moreover, we found that lowering MVK expression does not cause programmed cell death, as assessed by Propidium Iodide (PI) and Annex V (AnnV) staining. Using flow cytometry we found that untreated cells have 15.32 ± 1.32% double positive cell (positive for PI and AnnV), cells transfected with a “scrambled” (SCR) sequence have 14.08 ± 0.7% double positive, 8b transfected have 19.02 ± 0.96% and 8c transfected have 17.12 ± 1.04 double positive cells. The small differences in percentage are not significant with p < 0.05 (data not shown). We also tried to determine if the effect of lowering MVK expression could be more significant after LPS treatment, used as a trigger for inflammasome platform activation. We then examined caspase-1 activity in siRNA cells, and we found that LPS treatment increased caspase-1 activity with all the different transfections, i.e. “scrambled” siRNA or 8b or 8c, although to the same rate, demonstrating once more that lowering MVK protein content does not influence NALP3 activity (Additional file 1: Figure S1b).

**Figure 2:**
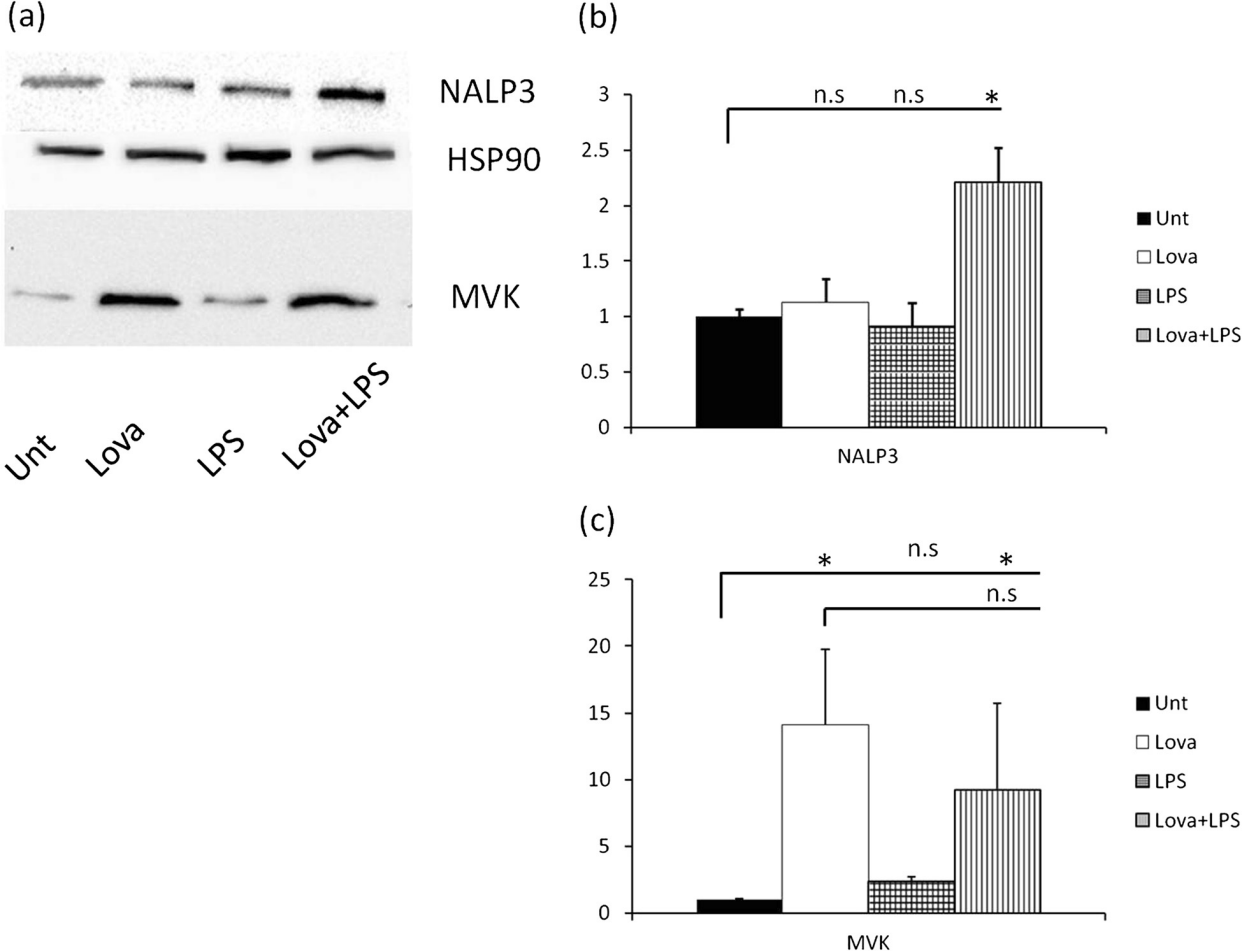
**Analysis of inflammasome components expression after LOVA+LPS treatment (a) representative western blot** **: untreated condition (UNT), lovastatin (LOVA) 10μM, LPS 0.5 ng/ml, LOVA + LPS double treatment. (b)** optical density calculation of MVK protein levels: n.s.: non significant differences * = p < 0.05 **(c)** optical density calculation of NALP3 protein levels: n.s.: non significant differences * = p < 0.05.

We also investigated the effect of Lovastatin treatment, alone or in combination with LPS, on MVK expression in BV-2 cells. Interestingly, the expression of MVK protein increased markedly both following stimulation with Lovastatin alone and with Lovastatin plus LPS (Figure 2a and b). Notably, only the combined treatment increased NALP3 expression (Figure 2a and c).

## Discussion

Our findings demonstrate that reduction in MVK protein up to 40% does not cause activation of the inflammasome and cell death. In one of the first work describing MKD, 3 different patients presented MVK protein levels lower than healthy controls [14]. It is thus possible that a “threshold” effect exists and below certain levels, the shortage of enzyme activity becomes pathological, as it could be hypothesized looking at our data on cell death (i.e. the observed reduction in enzyme content is not sufficient to cause cell death). Moreover, no improve in lowering MVK protein content has been observed also by combining the 2 siRNA used in this study (data not shown). It is thus possible that below certain levels of expression, cells do not survive. Although other experiments are needed to confirm this speculation, the previously cited work on KO mice might lend indirect support to this hypothesis [3]. In addition we propose an alternative explanations. It is possible that the activation of the Unfolded Protein Response (UPR)/autophagy might be caused by some *MVK* mutations that alter the protein structure, as seen by Houten and co-authors in 2002 [15]. However, this hypothesis remains untested and further works are needed to verify this mechanism.

It is also possible that the lack of inflammasome activation might be cell specific and thus other experiments would be required to test different cell types. Intriguingly, in a recent paper Van De Burg and co-authors [16] proposed that isoprenoid shortage leads to autophagy induction. The authors used simvastatin treatment in THP-1 and primary monocytes.

Finally, it should be also considered that UPR/autophagy response might be triggered by inhibiting the pathway, through statins treatment (or MVK mutations). In this line, using two different cell models (human airway mesenchimal cells and human atrial fibroblasts), Ghavami and co-authors demonstrated that simvastatin can activate UPR and autophagy and that this effect could be reverted by mevalonate administration but not by cholesterol supplementation, pointing to a crucial role of MVK and probably isoprenoid shortage in autophagy induction [17,18]. Furthermore, our results show that lovastatin treatment in BV-2 murine cells (Figure 2a and c) has a diametrically opposed effect to what observed in MKD patients, as shown by Houten et al. in 2002 [15], who found lower protein expression in fibroblast of patients carrying *MVK* mutations. Our results indeed show that lovastatin treatment results in a global increase of MVK protein content, up to fifteen times compared to untreated cells. Based on these findings, the lovastatin model should be amended, but also the siRNA model, at least in hour hands, was not able to mimic the pathologic features. Increase in MVK expression due to lovastatin, however, might be not sufficient to compensate for lower mevalonate acid content (substrate of MVK, probably reduced due to HMG-CoA blockade), also resulting in activation of a defective autophagic pathway [16-18]. So, we speculate that MVK protein levels could be important for NALP3 activation and the role of autophagy and UPR response could be pivotal in MKD and MA. However, as stated before, this remains just hypothesis and further experiments are needed to verify it.

## Additional file

Additional file 1: Figure S1. (a) caspase-1 activity, expressed as fold increase related to scrambled siRNA transfected cells (SCR), in cells transfected with 8b, 8c siRNA or left untreated (UNT). No statistical differences were observed. (b) caspase-1 activity, expressed as fold increase related to scrambled siRNA transfected cells (SCR), in cells transfected with 8b, 8c siRNA or left untreated (UNT). * = p < 0.05 in respect to each “mock” treated samples.
