# Supplementary figures and images for: Knockdown of MVK does not lead to changes in NALP3 expression or activation
Source: J Inflamm (Lond). 2015 Jan 31;12:7. doi: 10.1186/s12950-015-0048-5 (PMC4320511; doi:10.1186/s12950-015-0048-5)

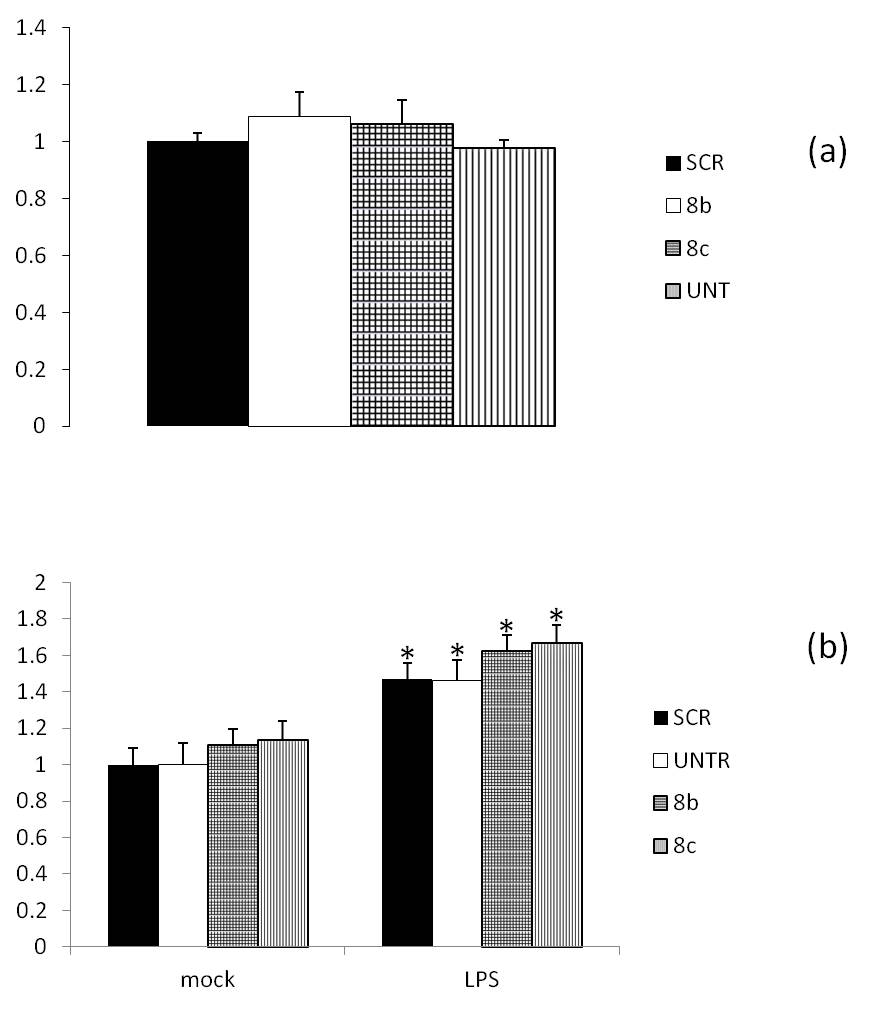

Supplement: Additional file 1: Figure S1. — (a) caspase-1 activity, expressed as fold increase related to scrambled siRNA transfected cells (SCR), in cells transfected with 8b, 8c siRNA or left untreated (UNT). No statistical differences were observed. (b) caspase-1 activity, expressed as fold increase related to scrambled siRNA transfected cells (SCR), in cells transfected with 8b, 8c siRNA or left untreated (UNT). * = p < 0.05 in respect to each “mock” treated samples. [file 12950_2015_48_MOESM1_ESM.jpeg]
